# Supplementary material for: Association between obesity and neurodevelopmental delay risk in children under five years: A study from Tumbes, Peru
Source: PLoS One. 2026 Mar 6;21(3):e0343815. doi: 10.1371/journal.pone.0343815 (PMC12965558; doi:10.1371/journal.pone.0343815)
Supplement: S1 Material — (DOCX) [file pone.0343815.s001.docx]

Supplementary Material 1. Informed Consent to participate in this study (Spanish and English versions).

**TITULO: OBESIDAD Y SU ASOCIACION CON TRASTORNOS DEL NEURODESARROLLO EN NIÑOS MENORES DE CINCO AÑOS UN ESTUDIO MULTICENTRICO. RED ESSALUD TUMBES 2021**

INVESTIGADORA: MIRIAM RODFELI ARREDONDO NONTOL

INSTITUCION: RED ASISTENCIAL TUMBES ESSALUD

**Propósito del estudio**

Estamos invitando a su hijo (a) a participar en un estudio donde se evaluará la presencia de trastornos del neurodesarrollo y obesidad, calidad de dieta, actividad física y anemia en población infantil de la zona. Este es un estudio desarrollado por investigadores de la Red Asistencial Essalud de Tumbes.

La obesidad puede ocasionar retardo en el desarrollo lo que puede estar agravado por una mala calidad de la dieta, poca actividad física y la presencia de anemia en los niños menores de 5 años.

**Procedimientos**

Si usted acepta que su hijo participe y su hijo decide participar en este estudio se le realizará lo siguiente:

1. Se le tomará una muestra de sangre de 5ml del antebrazo, esto es aproximadamente una cucharadita de té, para ver si tiene alteraciones en su sangre.

2. Se le realizará una evaluación del neurodesarrollo.

3. Se le tomara el peso y la talla para evaluar su estado nutricional.

4. Se le realizara una encuesta para evaluar su calidad de dieta y actividad física.

**Riesgos**

La toma de muestra de sangre es ligeramente dolorosa y existe un riesgo muy pequeño de que se pueda infectar si no se mantiene la higiene adecuada, además la toma de muestra del antebrazo puede ocasionar un pequeño hematoma (moretón) el cual desaparecerá en aproximadamente cinco días.

**Beneficios**

Su hijo (a) se beneficiará de una evaluación clínica y de los análisis sanguíneos para evaluar si existe alguna alteración.

Se le informará de manera personal y confidencial los resultados que se obtengan de los exámenes realizados. Los costos de todos los exámenes serán cubiertos por el estudio y no le ocasionarán gasto alguno.

**Costos y compensación**

No deberá pagar nada por la participación de su hijo (a) en el estudio. Igualmente, no recibirá ningún incentivo económico ni de otra índole, solo una compensación por gastos de transporte y/o un refrigerio por el tiempo brindado.

**Confidencialidad**

Nosotros guardaremos la información de su hijo (a) con códigos y no con nombres. Si los resultados de este seguimiento son publicados, no se mostrará ninguna información que permita la identificación de su hijo (a) o de otros participantes del estudio.

**Uso futuro de la información**

Deseamos conservar las muestras de su hijo (a) almacenándolas por 10 años. Estas muestras serán usadas para evaluar algunas pruebas diagnósticas. También usaremos esto para diagnosticar otras enfermedades.

Estas muestras solo serán identificadas con códigos.

Si usted no desea que las muestras de su hijo (a) permanezcan almacenadas ni utilizadas posteriormente, su hijo (a) aún puede seguir participando del estudio.

Además, la información de los resultados será guardada y usada posteriormente para estudios de investigación beneficiando al mejor conocimiento de la enfermedad y permitiendo la evaluación de medidas de control contra la obesidad y estilos de vida asi como la anemia .Se contará con el permiso del Comité Institucional de Ética en Investigación del Hospital Carlos Alberto Cortez Jiménez ,cada vez que se requiera el uso de las muestras y estas no serán usadas en estudios genéticos u otros estudios no relacionados al tema.

Autorizo a tener las muestras de sangre de mi hijo (a) almacenadas SI (..) NO (..)

**Derechos del participante:**

Si usted decide que su hijo (a) participe en el estudio, podrá retirarse de éste en cualquier momento, o no participar en una parte del estudio sin daño alguno. Si tiene alguna duda adicional, por favor pregunte al personal del estudio o llame a la Dra. MIRIAM ARREDONDO NONTOL, al teléfono 988930781

Si tiene preguntas sobre los aspectos éticos del estudio, o cree que su hijo (a) ha sido tratado injustamente puede contactar a la Dr. Nestor Purizaga Izquierdo miembro del Comité Institucional de Ética en Investigación del Hospital Carlos Alberto Cortez Jiménez de la Red Essalud Tumbes.

Una copia de este consentimiento informado le será entregada.

**DECLARACIÓN Y/O CONSENTIMIENTO**

Acepto voluntariamente que mi hijo (a) participe en este estudio, comprendo de las actividades en las que participará si ingresa al estudio, también entiendo que mi hijo (a) puede decidir no participar y que puede retirarse del estudio en cualquier momento.

---------------------------------- ---------------------------------------------

Nombres y Apellidos

Nombres y Apellidos Participante

Testigo (si el participante es analfabeto)

Fecha y Hora

--------------------------------------------------------------

Nombres y Apellidos investigador

Fecha y Hora: ……………………………………………………………………….

**TITLE: Association Between Obesity and Neurodevelopmental Disorders in Young Children: A Study from Tumbes, Peru**

RESEARCHER: MIRIAM RODFELI ARREDONDO NONTOL

INSTITUTION: ESSALUD TUMBES HEALTH CARE NETWORK

**Purpose of the Study**

We are inviting your child to participate in a study that will evaluate the presence of neurodevelopmental disorders and obesity, diet quality, physical activity, and anemia in the area's child population. This study is being conducted by researchers from the Essalud Healthcare Network in Tumbes.

Obesity can cause developmental delays, which can be aggravated by poor diet quality, limited physical activity, and anemia in children under 5 years of age.

**Procedures**

If you agree to your child's participation and your child decides to participate in this study, the following will be performed:

1. A 5 ml blood sample (approximately a teaspoon) will be taken from your forearm to check for blood abnormalities.

2. A neurodevelopmental evaluation will be performed.

3. Your child's weight and height will be measured to assess their nutritional status.

4. A survey will be conducted to assess the quality of their diet and physical activity.

**Risks**

Blood sampling is slightly painful, and there is a very small risk of infection if proper hygiene is not maintained. Blood sampling from the forearm may cause a small hematoma (bruise), which will disappear in approximately five days.

**Benefits**

Your child will benefit from a clinical evaluation and blood tests to assess any abnormalities.

You will be informed personally and confidentially of the results of the tests performed. The costs of all tests will be covered by the study and will not incur any additional costs to you.

**Costs and Compensation**

You will not be required to pay anything for your child's participation in the study. Likewise, your child will not receive any financial or other incentives, except compensation for transportation expenses and/or a snack for the time provided.

**Confidentiality**

We will store your child's information using codes, not names. If the results of this follow-up are published, no information that could identify your child or other study participants will be disclosed.

**Future Use of Information**

We wish to retain your child's samples for 10 years. These samples will be used to evaluate certain diagnostic tests. We will also use them to diagnose other diseases.

These samples will only be identified with codes.

If you do not wish your child's samples to remain stored or used later, your child can still participate in the study.

In addition, the information from the results will be stored and used later for research studies, benefiting from a better understanding of the disease and allowing for the evaluation of control measures against obesity and lifestyle, as well as anemia. Permission from the Institutional Research Ethics Committee of the Carlos Alberto Cortez Jiménez Hospital will be obtained each time the samples are required, and they will not be used in genetic or other studies unrelated to the topic.

I authorize the storage of my child's blood samples. YES (..) NO (..)

**Participant Rights:**

If you decide that your child participates in the study, you may withdraw at any time or not participate in any part of the study without any harm. If you have any additional questions, please ask the study staff or call Dr. Miriam Arredondo Nontol at 988930781.

If you have questions about the ethical aspects of the study, or believe that your child has been treated unfairly, you may contact Dr. Nestor Purizaga Izquierdo, a member of the Institutional Research Ethics Committee of the Carlos Alberto Cortez Jiménez Hospital of the Essalud Tumbes Network.

A copy of this informed consent will be given to you.

**DECLARATION AND/OR CONSENT**

I voluntarily agree that my child may participate in this study. I understand the activities my child will participate in if enrolled in the study. I also understand that my child may decide not to participate and may withdraw from the study at any time.

---------------------------------- ---------------------------------------------

First and Last Names

Participant's First and Last Names

Witness (if the participant is illiterate)

Date and Time --------------------------------------------------

Researcher's First and Last Names

Date and Time: ……………………………………………..……………………………….
