# Supplementary material for: Association between obesity and neurodevelopmental delay risk in children under five years: A study from Tumbes, Peru
Source: PLoS One. 2026 Mar 6;21(3):e0343815. doi: 10.1371/journal.pone.0343815 (PMC12965558; doi:10.1371/journal.pone.0343815)

Supplementary Material 2. Complete Directed Acyclic Graph of this study

1. **Model Presentation**

A Directed Acyclic Graph (DAG) was constructed to represent the hypothetical causal relationships between childhood obesity in children under five years of age (exposure variable) and **Neurodevelopmental Delay Risk** (outcome variable). The DAG was developed using the DAGitty.net platform and was informed by recent scientific literature addressing perinatal, maternal, and environmental factors associated with early neurological development.

This graphical model served as a theoretical tool to identify which variables should be included in the multivariate analysis, aiming to avoid adjustment for mediators or collider variables and to accurately estimate the total effect of obesity on the outcome.

1. **Variables included in the DAG**

| **Variable** | **Role in DAG** | **Justification** |
| --- | --- | --- |
| Childhood obesity | Main exposure | Observational studies have shown that obesity affects multiple areas of child development (11,12) |
| **Neurodevelopmental Delay Risk** | Outcome | Neurological and cognitive impairments that may originate from biological and environmental factors during early childhood (72) |
| Maternal age | Confounder | Associated with increased risk of childhood obesity and with neurodevelopmental outcomes (73,74) |
| Maternal education level | Confounder | Influences parenting practices, child feeding, and stimulation. Associated with both obesity and cognitive development (75,76). |
| Maternal Knowledge of complementary feeding | Confounder | Directly impacts the likelihood of childhood obesity and diet quality, a factor linked to neurodevelopment (54,77) |
| Physical activity compliance | Confounder | Associated with prevention of childhood obesity and indirectly stimulates child development (51,78). |
| Anemia | Mediator | Childhood obesity may alter iron bioavailability and contribute to anemia, which in turn has been associated with cognitive and motor delays (79) |

1. **Selection of the Minimal Sufficient Adjustment Set**

Based on the DAG analysis, the minimal sufficient set of variables was identified for adjustment in the multivariate model to block confounding pathways between childhood obesity and neurodevelopmental disorders. The selected variables were:

- Maternal age
- Maternal education level
- Maternal knowledge of complementary feeding
- Physical activity compliance

Anemia was excluded from the adjustment set, as it was considered a mediator in the direct causal pathway between the exposure and the outcome. Including it could introduce overadjustment bias and distort the estimation of the total effect.

1. **Directed Acyclic Graph (DAG)**


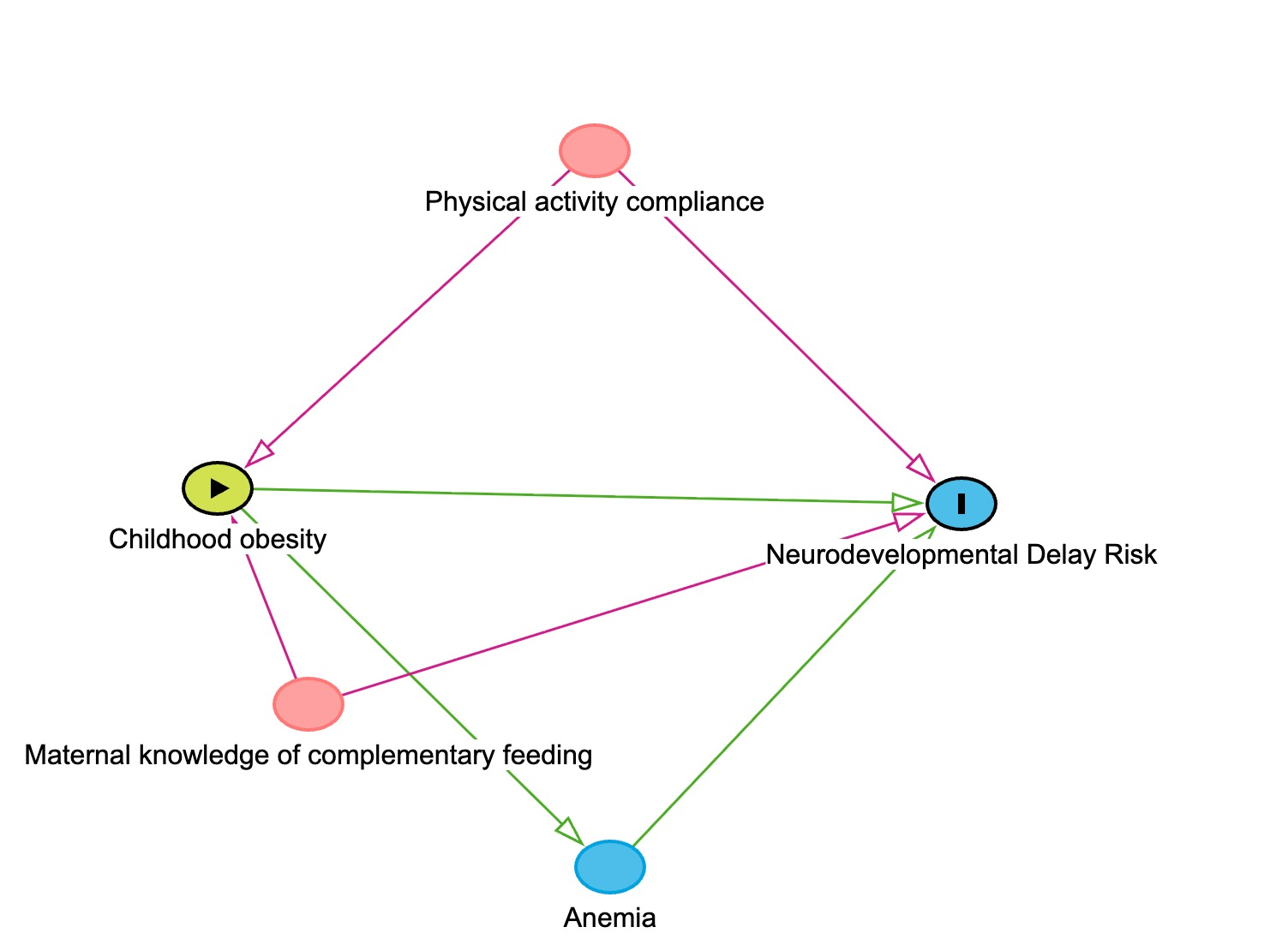

Supplement: S2 Material — (DOCX) [file pone.0343815.s002.docx]
