# Supplementary material for: Association between obesity and neurodevelopmental delay risk in children under five years: A study from Tumbes, Peru
Source: PLoS One. 2026 Mar 6;21(3):e0343815. doi: 10.1371/journal.pone.0343815 (PMC12965558; doi:10.1371/journal.pone.0343815)
Supplement: Attachment — Submitted filename: Respuesta a revisores.docx [file pone.0343815.s005.docx]

Response to Reviewers’ Comments

Association Between Obesity and Neurodevelopmental Disorders in Young Children: A Study from Tumbes, Peru

PONE-D-25-30834

Plos one

A. ACADEMIC EDITOR

| COMMENTS | RESPONSE | MANUSCRIPT CHANGES |
| --- | --- | --- |
| Conceptual aspects and terminology | | |
| Standardize the terminology related to the outcome, avoiding the interchangeable use of “neurodevelopmental delay” and “developmental disorders” | We agree with this  observation. We have standardized the terminology related to the outcome throughout the manuscript, avoiding the interchangeable use of terms. Specifically, we consistently use the term “risk of neurodevelopmental delay” to refer to the outcome assessed through a developmental screening instrument, clearly distinguishing it from clinically diagnosed neurodevelopmental disorders.  Additionally, to ensure full conceptual coherence, we have revised the manuscript title to more accurately reflect that the study evaluates the risk of neurodevelopmental delay identified through screening, rather than formally diagnosed neurodevelopmental disorders. All sections of the manuscript (Abstract, Methods, Results, Discussion, tables, and figures) were reviewed and updated accordingly to align with this change | Revised throughout the manuscript. |
| Revise the Discussion and Conclusion to avoid causal inferences, ensuring that the language reflects associations observed in a cross-sectional study. | The Discussion and Conclusion sections were comprehensively revised to ensure full coherence with the study’s cross-sectional design. All causal or prescriptive language was removed or reformulated using associative terminology (e.g., “was associated with,” “showed a higher probability,” “may reflect,” “has been described”). Interpretations were reframed to emphasize associations rather than causal relationships. In addition, the Conclusion was modified to highlight the observational nature of the findings, the probabilistic use of the nomogram, and the need for external validation prior to broader application. | Revised in the Discussion (lines 517–741) and Conclusions (lines 743–776). |
| Include an explicit discussion of the possibility of reverse causality, particularly in the relationships between physical activity, obesity, and developmental delay. | We thank the reviewer for this important observation. In response, we have explicitly incorporated into the Discussion section a reflection on the possibility of reverse causality in the observed associations between physical activity, obesity, and the risk of neurodevelopmental delay. These relationships are acknowledged to be potentially bidirectional, as lower levels of physical activity and obesity may be associated with neurodevelopmental delay, while neurodevelopmental delay itself may also lead to reduced physical activity levels and an increased risk of obesity. Furthermore, we emphasize that, due to the cross-sectional design of the study, it is not possible to establish the temporal directionality of these associations. | Revised in the Discussion section (lines 603–619). |
| 2) Study design, sample, and ethical aspects | | |
| Clearly specify the inclusion and exclusion criteria of the sample. | We thank the reviewer for this observation. In response, the Study Design and Population section was revised to clearly specify the inclusion and exclusion criteria of the sample, which are now presented under a dedicated subheading. | Revised in the Methods section (Study Design) (lines 205–215). |
| Indicate whether a sample size calculation was performed; if not, clarify whether the sample was defined as census-based or consecutively recruited. | We thank the reviewer for this observation. A sample size calculation was performed during the study planning phase based on the expected prevalence of the outcome of interest. **However, the final sample size was determined by consecutive recruitment of all eligible children attending the participating healthcare facilities during the study period.**  During the conduct of the study, participants were included through consecutive recruitment, enrolling all eligible children under five years of age who attended the participating facilities and whose parents or legal guardians provided informed consent. This clarification has been incorporated into the Study Design and Population section of the manuscript. | Revised in the Methods section(lines 188-193) |
| Justify the selection of the age group (<5 years) and clarify whether children with Autism Spectrum Disorder were included.  . | We thank the reviewer for this observation. The selection of children under five years of age is justified by the fact that early childhood represents a critical period of neurodevelopment, highly sensitive to nutritional, metabolic, and behavioral factors, and also corresponds to the age range for which the *Evaluación del Desarrollo Infantil* (EDI) instrument has been validated. This justification has been incorporated into the Methods section.  In addition, the exclusion criteria have been clarified to specify that children with prior clinical diagnoses of neurodevelopmental disorders, including Autism Spectrum Disorder, were not included in the study. | Revised in the Methods section and in the Inclusion and Exclusion Criteria (lines 199–215). |
| Clarify whether the ethics approval covered any extensions of the data collection period  . | We thank the reviewer for this observation. The study received initial ethical approval in August 2021 from the Institutional Research Ethics Committee of the EsSalud Tumbes Healthcare Network. Ethical approval was subsequently renewed on an annual basis through official certifications issued in 2022, 2023, and 2024, thereby ensuring ethical oversight throughout the entire data collection period. This clarification has been incorporated into the Methods section of the manuscript. | Revised in the Ethical Approvals section (lines 218–233). |
| Expand the discussion on the limited representativeness of the sample (restricted to the EsSalud network), including potential impacts on prevalence estimates and effect measures, as well as whether post-stratification weighting or sensitivity analyses were performed. | We thank the reviewer for this comment. The Discussion section has been expanded to explicitly address the limited representativeness of the study sample, which was restricted to children receiving care within the EsSalud healthcare network. As EsSalud primarily serves formally employed populations and their dependents, the study population may differ from uninsured children, those attending Ministry of Health facilities, or populations in more rural or socioeconomically disadvantaged settings.  This restriction may primarily affect prevalence estimates, which should therefore be interpreted with caution, as children with regular access to healthcare services may differ in nutritional status, health-seeking behavior, and early detection of developmental risk. With respect to effect measures, while selection into the EsSalud system may influence baseline risk, the internal validity of the observed associations is less likely to be substantially compromised, assuming that exposure–outcome relationships operate similarly within this healthcare setting.  Post-stratification weighting and sensitivity analyses were considered; however, these approaches were not performed due to the absence of population-level reference distributions for the EsSalud pediatric population and the cross-sectional design of the study. These considerations are now explicitly stated in the Limitations section of the manuscript. | Revised in the Limitations section (lines 683–695). |
| 3) Measurement and operationalization of variables | | |
| 3.1) Physical activity: | | |
| Provide a detailed description of the data collection instrument (questionnaire and specific questions used). | We thank the reviewer for this observation. The Methods section has been expanded to provide a more detailed description of the instrument used to assess physical activity. Physical activity was evaluated using a structured questionnaire administered to caregivers, specifically developed for this study and directly based on the World Health Organization’s physical activity recommendations for children under five years of age. The instrument classified compliance with these recommendations according to age groups and was used as a categorical variable in the analysis. In addition, it is acknowledged that the questionnaire does not constitute a formally validated scale, which has been identified as a limitation of the study. | Revised in the Methods section (Physical Activity Compliance) (lines 248–268). |
| - Justify the dichotomization of the variable and discuss the associated loss of information. | We thank the reviewer for this observation. The dichotomization of the physical activity variable was performed to classify compliance with the World Health Organization’s age-specific recommendations, prioritizing clinical interpretability and comparability across age groups in early childhood. In addition, a reflection on the potential loss of information associated with this methodological decision has been incorporated into the Discussion section, acknowledging that gradients of physical activity intensity or duration are not captured, which represents a limitation of the study. | Revised in the Discussion (Limitations subsection) (lines 708–720). |
| Explicitly describe how WHO recommendations were operationalized across different age groups,  particularly among children under one year of age. | We thank the reviewer for this observation. The Methods section has been expanded to explicitly describe how the World Health Organization’s physical activity recommendations were operationalized according to age groups. In particular, the criterion applied to children under one year of age was detailed, based on engagement in active play and time spent in the prone position (“tummy time”) during the day, in accordance with WHO recommendations. In addition, the physical activity thresholds applied for the 1–2 and 3–4 year age groups were specified. | Revised in the Physical Activity Compliance section (lines 248–268). |
| Critically discuss the high prevalence of compliance observed and the potential for social desirability bias. | We thank the reviewer for this observation. The Discussion section has been expanded to critically address the high prevalence of compliance with physical activity recommendations observed in the study. In particular, we discuss the potential impact of social desirability bias associated with the use of caregiver-reported information, as well as the influence of dichotomous classification based on international guideline thresholds. These considerations have been incorporated as part of the study’s limitations. | Revised in the Limitations section (lines 707–712). |
| 3.2) Anemia: | | |
| Specify the data source (laboratory measurements or caregiver report). | We thank the reviewer for this observation. The Methods section now specifies that anemia was determined exclusively through laboratory measurements, using hemoglobin concentration obtained from venous blood samples, and that no caregiver-reported information was used for its classification. | Revised in the Methods section (Anemia Assessment subsection) (lines 270–275). |
| Describe the cutoff values used, any altitude adjustments (if applicable), and the references adopted. | We thank the reviewer for this observation. The Methods section now specifies the cutoff points used for the definition of anemia, based on the Clinical Practice Guideline of the Peruvian Ministry of Health (MINSA). Specifically, anemia was defined as hemoglobin <13.5 g/dL in children younger than 2 months, <9.5 g/dL in children aged 2 to 5 months, and <11.0 g/dL in children aged 6 to 59 months.  In addition, it was clarified that, for analytical purposes, anemia was operationalized as a dichotomous variable (presence/absence) across all age groups, in order to ensure consistency and comparability in the analysis.  Given that the study was conducted in Tumbes, a coastal region located at sea level, no altitude adjustment was applied to hemoglobin values. All definitions and cutoff points used were based on the official recommendations of the Peruvian Ministry of Health. | Revised in the Methods section (Anemia Assessment) (lines 276–285). |
| 3.3) Maternal knowledge of complementary feeding: | | |
| Detail the scoring system and cutoff points used | We thank the reviewer for this observation. The Methods section now details the scoring system used for the maternal knowledge questionnaire on complementary feeding. Each correct response was awarded one point, whereas incorrect or “do not know” responses received zero points. The total score was obtained by summing the correct responses and was used to classify maternal knowledge into categories (low, moderate, and good), which were subsequently used in the statistical analysis. | Revised in the Methods section (Maternal Knowledge of Complementary Feeding) (lines 286–299). |
| Create specific subsections in the Methods for physical activity and anemia to improve clarity and reproducibility. | We thank the reviewer for the suggestion. In response, the Methods section was reorganized to incorporate specific subsections entitled “Physical Activity Assessment” and “Anemia Assessment,” in which the measurement procedures, operationalization, and criteria used for each variable are described in a detailed and separate manner, in order to improve the clarity and reproducibility of the study. | Revised in the Methods section (lines 179–428). |
| 4) Missing data and sociodemographic variables | | |
| - Report the presence and proportion of missing data. | *We thank the reviewer for this observation. Of the 445 initial records, 3 were excluded prior to analysis due to ineligibility (n = 2) or duplication (n = 1). Among the 442 eligible participants, 11 children (2.5%) had missing data in at least one key variable, corresponding to hemoglobin (n = 10) or the maternal questionnaire (n = 1), as detailed in Table 1.*  *Given the low proportion of missing data and the absence of systematic patterns, multivariable analyses were conducted using a complete-case approach, resulting in a final analytical sample of 431 children. No imputation methods were applied.* | Revised in the Methods section (Statistical Analysis) (lines 349–356). |
| Describe how missing data were handled in the analysis. | We thank the reviewer for this observation. Missing data were assessed prior to analysis. Records with incomplete information in key variables were excluded from the multivariable analysis, and a complete-case analysis was performed. No imputation methods were applied, as the proportion of missing data was low and no systematic patterns of missingness were identified. | Revised in the Methods section (Statistical Analysis) (lines 349–356). |
| Systematically present all sociodemographic variables collected and ensure consistency between the Methods, Results, and Tables. | We thank the reviewer for this observation. All sociodemographic variables collected (child age and sex, maternal age, and maternal educational level) are now presented in a systematic and consistent manner across the Methods and Results sections, as well as in the corresponding tables. In addition, the consistency of variable naming and categorization throughout the manuscript was verified, in accordance with recommendations for the reporting of observational studies. | Revised in the Methods section (lines 300–306) and the Results section (lines 433–440). |
| 5) Statistical analysis and modeling: | | |
| Clarify the analytical objective of the study (explanatory vs. predictive) and align methodological decisions accordingly. | We thank the reviewer for this important comment. The primary analytical objective of the study is explanatory, aimed at estimating the association between childhood obesity and neurodevelopmental delay risk. Accordingly, we used an ordinal logistic regression model guided by a causal framework (DAG) to control for confounding and avoid overadjustment.  A nomogram and online calculator were developed as a secondary, applied objective to facilitate individual probabilistic risk estimation based on the explanatory model, rather than to optimize predictive performance. We have clarified this distinction in the Objectives and Statistical Analysis sections. | Revised in the Introduction (lines 169–175) and in the Methods section (Statistical Analysis) (lines 319–323). |
| Justify the exclusion of anemia based on the DAG in the context of a predictive model, or consider alternative analytical approaches. | We thank the reviewer for this insightful comment. In our study, anemia was included in the initial multivariable ordinal logistic regression model as part of the explanatory analysis, based on the causal assumptions defined in the Directed Acyclic Graph (DAG). This approach allowed us to evaluate its potential confounding role in the association between childhood obesity and neurodevelopmental delay risk.  However, anemia was not significantly associated with the outcome in the multivariable model and did not meaningfully contribute to model discrimination. Given that the primary analytical objective of the study was explanatory, and that the predictive component (nomogram and online calculator) was developed as a secondary, applied objective derived from the explanatory model, we opted to exclude anemia from the final nomogram to ensure model parsimony, interpretability, and clinical usability.  This decision is consistent with recommended practices in clinical prediction modeling, where variables are selected not only based on theoretical relevance but also on their contribution to predictive performance and stability.(Feng2024) We have clarified this distinction between the explanatory model and the applied predictive tool in the Methods and Statistical Analysis sections.  We agree that alternative analytical approaches—such as retaining anemia in a purely predictive model or applying penalized regression techniques—could be explored in future studies with larger samples and longitudinal designs. These possibilities have been acknowledged as avenues for further research. | Revised in the Methods section (Model Training and Validation) (lines 373–395), the Results section (Multivariate Ordinal Regression Analysis) (lines 494–496), and the Discussion (lines 633–636). |
| Discuss the impact of excluding potential mediators on model performance. | Thank you for this comment. In our DAG-based explanatory framework, anemia lies downstream of childhood obesity and thus represents a potential mediator (obesity → anemia → neurodevelopmental delay risk). Excluding this mediator from the primary model preserves estimation of the total effect of obesity and avoids overadjustment. We acknowledge that including mediators can sometimes improve apparent predictive performance by capturing downstream information; however, this comes at the cost of causal interpretability and may attenuate the estimated association of obesity with the outcome. Therefore, we excluded anemia from the explanatory model, while noting that mediators could be considered in a purely predictive model depending on the intended use. | Revised in the Discussion (Limitations subsection) (lines 736–742). |
| Revisit the interpretation of the role of physical activity, explicitly acknowledging the possibility of a bidirectional association with the outcome. | We thank the reviewer for this comment and agree that the interpretation of the role of physical activity should explicitly acknowledge the possibility of a bidirectional association with neurodevelopmental outcomes.  As previously requested, we had already incorporated an explicit discussion of reverse causality in the Discussion section, emphasizing that lower physical activity levels may both contribute to, and result from, neurodevelopmental vulnerability, particularly given the cross-sectional nature of the study.  In response to the present comment, we have further refined the interpretation of physical activity in the Discussion to avoid any implicit unidirectional or causal framing. Specifically, we clarified that physical activity should be interpreted as a correlational marker rather than a determinant of neurodevelopmental delay risk, explicitly highlighting the potentially bidirectional nature of this association when discussing underlying mechanisms. These revisions reinforce that the observed associations may reflect reciprocal pathways rather than a single directional effect and are fully consistent with the limitations inherent to the study design. | Revised in the Discussion section (lines 604–620). |
| 6) Results, tables, and presentation: | | |
| - Ensure that all tables cited in the text are included and correctly numbered | We thank the reviewer for this comment. We have carefully verified that all tables cited in the text are included in the manuscript and are correctly numbered. All in-text table citations correspond accurately to the appropriate tables. | **Revised in the Tables section (lines 1187–1258).** |
| Make tables self-explanatory by including cutoff values for obesity, anemia, and physical activity in table legends or notes. | We thank the reviewer for this suggestion. We have revised the table legends and notes to include the cutoff values used to define childhood obesity, anemia, and physical activity, ensuring that all tables are fully self-explanatory without reference to the main text. | **Revised in the Tables section (lines 1187–1258).** |
| Correct inconsistencies and typographical errors (e.g., missing spaces before citations). | We thank the reviewer for this comment. We have carefully revised the manuscript to correct typographical errors and minor inconsistencies, including spacing and formatting issues before citations, to improve overall clarity and consistency. | Revised throughout the manuscript. |
| 7) Discussion and applicability of the nomogram: | | |
| - Revise the opening paragraph of the Discussion to avoid repetition of the study objective and excessive description of results. | We thank the reviewer for this suggestion. We have revised the opening paragraph of the Discussion to avoid repetition of the study objective and excessive description of results, shifting the focus toward interpretation and contextualization of the main findings. | Revised in the Discussion section (lines 518–525). |
| Expand the discussion on the contexts in which the nomogram may be applied and its external validity, particularly in populations outside insured healthcare networks and across different socioeconomic settings. | We expanded the Discussion to explicitly address the contexts in which the nomogram may be applied, particularly in populations outside insured healthcare networks, and to discuss considerations regarding external validity and generalizability across different socioeconomic settings | Revised in the Discussion section (lines 650–670). |
| 8) References | | |
| Review and standardize all references, ensuring completeness, accuracy, and correct correspondence between in-text citations and the reference list. | We thank the reviewer for this comment. We have carefully reviewed and standardized all references to ensure completeness and accuracy. All in-text citations were cross-checked against the reference list to confirm correct correspondence, and the reference list was revised to ensure consistent formatting and inclusion of all required bibliographic details according to the journal’s guidelines. | Revised in the References section (lines 782–1117). |

B.JOURNAL REQUIREMENTS:

| COMMENT | RESPONSE | MANUSCRIPT CHANGES |
| --- | --- | --- |

| When submitting your revision, we need you to address these additional requirements.  1. Please ensure that your manuscript meets PLOS ONE's style requirements, including those for file naming. The PLOS ONE style templates can be found at  <https://journals.plos.org/plosone/s/file?id=wjVg/PLOSOne_formatting_sample_main_body.pdf> and  [https://journals.plos.org/plosone/s/file?id=ba62/PLOSOne_formatting_sample_title_authors_affiliations.pdf](https://track.editorialmanager.com/CL0/https:%2F%2Fjournals.plos.org%2Fplosone%2Fs%2Ffile%3Fid=ba62%2FPLOSOne_formatting_sample_title_authors_affiliations.pdf/1/010f019ba1b8ccc5-be39cfe9-59c2-466c-b10a-9f9865de426c-000000/M1gxnnjs-gQtJiEJ0jcE3U7G965KS2jl1zH3iCu03dE=245) | We have revised the manuscript and all submission files to ensure full compliance with PLOS ONE’s style requirements, including formatting, structure, and file naming conventions, following the official PLOS ONE templates for the main body and for the title, authors, and affiliations. | Revised throughout the manuscript. |
| --- | --- | --- |
| 2. We note that the grant information you provided in the ‘Funding Information’ and ‘Financial Disclosure’ sections do not match. | Thank you for bringing this to our attention. We have reviewed the information provided in the submission system and ensured that the ‘Funding Information’ and ‘Financial Disclosure’ fields are now fully consistent. The correct funding source has been accurately reported in both sections. | Updated in the Funding Information and Financial Disclosure fields in the submission system. |
| When you resubmit, please ensure that you provide the correct grant numbers for the awards you received for your study in the ‘Funding Information’ section. | Thank you for this comment. We have reviewed the Funding Information section and clarified that the support received through the IETSI–EsSalud Mentoring Program was not associated with a specific grant number. This information has now been explicitly stated in the Funding Information section. | Funding information was clarified in the submission system to indicate that no specific grant number applies. |
| 3. Thank you for stating the following financial disclosure:  “This study was partially funded by the Institute for Health Technology Assessment and Research (IETSI), ESSALUD, through its Mentoring Program.”  Please state what role the funders took in the study.  If the funders had no role, please state: "The funders had no role in study design, data collection and analysis, decision to publish, or preparation of the manuscript."  If this statement is not correct you must amend it as needed.  Please include this amended Role of Funder statement in your cover letter; we will change the online submission form on your behalf. | Thank you for this request. We confirm that the funders had no role in the study design, data collection and analysis, decision to publish, or preparation of the manuscript. As requested, this amended Role of the Funder statement has been included in the cover letter. | Added to the cover letter, as requested by the editor. |
| 4. Please note that funding information should not appear in any section or other areas of your manuscript. We will only publish funding information present in the Funding Statement section of the online submission form. Please remove any funding-related text from the manuscript. | Thank you for the clarification. We have removed all funding-related text from the manuscript, as requested. Funding information is now reported exclusively in the Funding Statement section of the online submission form. | All funding-related text has been removed from the manuscript. Funding information is now reported exclusively in the Funding Statement section of the online submission form, as requested. |
| 5. Your ethics statement should only appear in the Methods section of your manuscript. If your ethics statement is written in any section besides the Methods, please move it to the Methods section and delete it from any other section. Please ensure that your ethics statement is included in your manuscript, as the ethics statement entered into the online submission form will not be published alongside your manuscript. | Thank you for the clarification. We have moved the ethics statement to the Methods section of the manuscript and included it under a dedicated “Ethics approval” subsection, removing it from any other section, as requested. | Revised in the Methods section under a dedicated “Ethics approval” subsection; the ethics statement was removed from all other sections. |
| 6. If the reviewer comments include a recommendation to cite specific previously published works, please review and evaluate these publications to determine whether they are relevant and should be cited. There is no requirement to cite these works unless the editor has iindicated otherwise | We acknowledge the reviewer’s comment and confirm that all references included in the manuscript were carefully selected based on their direct relevance to the study objectives. | Revised throughout the manuscript. |

C. REVIEW COMMENTS TO THE AUTHOR

Reviewer #1:

| COMMENT | RESPONSE | MANUSCRIPT CHANGES |
| --- | --- | --- |
| 1.Standardize the terminology.At times, the terms “neurodevelopmental delay” and “developmental disorders” are used interchangeably. Consider standardizing terminology to improve clarity | We agree with the reviewer’s observation. This issue had also been raised by the Academic Editor and has been fully addressed in the revised manuscript. The terminology related to the outcome has been standardized throughout the text, avoiding the interchangeable use of “neurodevelopmental delay” and “developmental disorders.”  Specifically, we consistently use the term “risk of neurodevelopmental delay” to refer to the outcome assessed through a developmental screening instrument, clearly distinguishing it from clinically diagnosed neurodevelopmental disorders.  In addition, to ensure full conceptual coherence, the manuscript title was revised to accurately reflect that the study evaluates *risk identified through screening* rather than formally diagnosed disorders. All sections of the manuscript (Abstract, Methods, Results, Discussion, tables, and figures) were reviewed and updated accordingly. | Revised throughout the manuscript. |
| 2.Ensure all references are complete and accurate and cited in parentheses (e.g., (8), (46–49)) corresponding accurately to a complete reference list, particularly those supporting prevalence figures and previous nomogram applications | We appreciate the reviewer’s comment. All references have been carefully reviewed to ensure completeness, accuracy, and correct formatting. Citations throughout the manuscript were verified to correspond accurately to the reference list and are consistently presented in parentheses using numerical citation style (e.g., (8), (46–49)).  In particular, references supporting prevalence estimates and prior applications of nomograms were cross-checked for accuracy and completeness, including author names, publication year, journal details, and page or article numbers. The reference list has been updated accordingly to ensure full consistency with journal requirements. | Revised throughout the manuscript. |
| 3.Correct minor typographical issues (e.g., missing spaces before reference citations). | We thank the reviewer for this observation. We have carefully reviewed the entire manuscript to identify and correct minor typographical issues, including missing spaces before reference citations and other formatting inconsistencies.  Following this thorough proofreading process, we confirm that all such issues have been addressed, and the manuscript now complies with the journal’s formatting and citation style requirements. | Revised throughout the manuscript. |
| 4.Check table numbering and ensure all tables referenced in the text are included in the submission. | We thank the reviewer for this comment. We carefully checked the numbering of all tables and verified that every table cited in the text is correctly numbered and fully included in the submitted manuscript files.  This review confirmed consistency between in-text table citations, table numbering, and the tables provided in the submission. Therefore, no additional changes were required. | **Revised in the Tables section (lines 1187–1258).** |
| 5.Clarify whether ethics approval covered any extensions to the study period | Thank you for this comment. The ethics approval covered all extensions of the data collection period. Initial approval was obtained in 2021 and was renewed annually in 2022, 2023, and 2024, ensuring continuous ethical oversight throughout the entire study period. This has been clearly specified in the Ethics section of the manuscript. | Revised in the Methods section (Ethics Approval) (lines 217–233). |
| 6.Provide operational details for physical activity compliance, anemia diagnosis (including any altitude adjustments), and scoring thresholds for complementary feeding knowledge | We thank the reviewer for this comment. In response, we have expanded the Methods section to provide detailed operational definitions for physical activity compliance, anemia diagnosis, and maternal knowledge of complementary feeding.  Physical activity compliance is now described using age-specific criteria derived from World Health Organization recommendations, including explicit operationalization for children under one year of age. Physical activity was classified based on caregiver-reported data and dichotomized according to adherence to recommended thresholds.  Anemia diagnosis is clearly defined using laboratory-measured hemoglobin concentrations and age-specific cut-off values established by national clinical practice guidelines. As the study was conducted in a coastal region at near sea level, no altitude adjustment of hemoglobin values was applied, and this has been explicitly stated.  Maternal knowledge of complementary feeding is now described with a detailed scoring system, including the total score range and predefined cutoff points used to classify knowledge levels. These clarifications were incorporated to improve transparency, reproducibility, and methodological clarity. | Revised in the Methods section (lines 234–306). |
| 7.The sample was drawn entirely from insured patients (EsSalud network), which may limit applicability to uninsured or rural populations. This limitation is mentioned briefly, but more detail is needed on how this might affect both prevalence estimates and effect measures, and whether any post-stratification weighting or sensitivity analyses were considered | We appreciate the reviewer’s observation. The study sample was drawn entirely from insured children within the EsSalud healthcare network, which may limit the applicability of the findings to uninsured populations, children receiving care in Ministry of Health facilities, or those living in rural settings. This limitation has been addressed in greater detail in the revised Discussion and Limitations sections.  Specifically, this restriction may influence prevalence estimates of obesity, anemia, and risk of neurodevelopmental delay, as these outcomes may differ in populations with less consistent access to healthcare services. In contrast, the estimated effect measures are less likely to be substantially biased, provided that the underlying exposure–outcome relationships are similar within the study population, although some degree of selection-related difference cannot be fully excluded.  Post-stratification weighting was not applied because the population-level distributions required for valid weighting were not available for the EsSalud pediatric population. Additionally, sensitivity analyses addressing differential healthcare access were considered but were not feasible given the cross-sectional design and the lack of comparable external reference data. These points are now clearly acknowledged in the manuscript. | Revised in the Limitations section (lines 685–696). |
| 8.Although the manuscript acknowledges its cross-sectional design, parts of the discussion use language suggestive of causality (e.g., stating that obesity “increases” the risk of developmental delay). The authors should revise the text to reflect associations rather than causal effects. Additionally, the potential for reverse causation—such as developmental delay leading to reduced physical activity and subsequent obesity—should be explicitly addressed. | We thank the reviewer for this important observation. In response, we carefully revised the Discussion and Conclusion sections to ensure that all language reflects associations rather than causal effects, consistent with the cross-sectional design of the study.  Additionally, we have incorporated an explicit discussion of the possibility of reverse causation, particularly in the relationships between physical activity, obesity, and neurodevelopmental delay. We now acknowledge that neurodevelopmental vulnerability may precede and influence lower physical activity levels and increased sedentary behavior, which could subsequently contribute to obesity, rather than obesity or low physical activity acting as unidirectional causes.  These clarifications have been incorporated to avoid causal interpretations and to emphasize the bidirectional and correlational nature of the observed associations. | Revised throughout the manuscript, with specific changes in the Discussion section (lines 604–620) and the Conclusions section (lines 744–777). |
| 9.Any specific reason to select the sample size under five years of age ,as nothing is mentioned in manuscript.Children above 5 are also at high risk of obesity due to the spreading epidemic related to sedentary life style and poor dietrery habits. | We thank the reviewer for this comment. The rationale for focusing on children under five years of age has been clarified in the Methods section. Early childhood represents a critical window for neurodevelopment, during which developmental trajectories are particularly sensitive to nutritional, metabolic, and behavioral factors.  In addition, the selected age range corresponds to the validated target population of the *Evaluación del Desarrollo Infantil* (EDI), which is designed for use in children from birth to 59 months. This ensured methodological consistency between the study objectives and the developmental assessment instrument.  While children older than five years are also at increased risk of obesity, particularly in the context of sedentary lifestyles and unhealthy dietary habits, the present study was specifically designed to examine associations between obesity-related factors and early neurodevelopmental vulnerability, which is most appropriately assessed during the first five years of life. For this reason, older children were outside the scope of the present analysis. | Revised in the Methods section (Study Design and Population) (lines 188–204). |
| 10.Were children with Autism included in study? | Children with previously diagnosed Autism Spectrum Disorder were excluded according to the predefined exclusion criteria. | Revised in the Methods section (Exclusion Criteria) (lines 211–215). |

Reviewer #2:

The manuscript addresses a topic of significant public health interest: the association between childhood obesity and neurodevelopment. The authors are to be acknowledged for selecting this subject, particularly within the challenging context of the "double burden" of malnutrition. Furthermore, the study’s inclusion of physical activity as a focal point is noteworthy, as it appropriately draws attention to the role of movement in early childhood development beyond its function in weight management. However, despite these conceptual strengths, a detailed review of the manuscript reveals significant methodological limitations that currently compromise the validity of the findings. Specific concerns regarding study design, variable measurement, and statistical analysis are detailed below.

| COMMENT | RESPONSE | MANUSCRIPT CHANGES |
| --- | --- | --- |
| First, a fundamental methodological conflict exists between the study's dual goals of explaining an association and building a predictive tool. The authors employed a DAG to exclude "Anemia" on the basis that it acts as a "mediator". While excluding mediators is standard practice for establishing the isolated causal effect of obesity, it is often counterproductive for a predictive model. In prediction scenarios, mediators are frequently the strongest predictors and should be retained to maximize model accuracy. Consequently, the exclusion of anemia likely contributes to the model's suboptimal performance. | We thank the reviewer for this insightful methodological comment. We agree that explanatory and predictive objectives rely on different modeling principles and that, in purely predictive settings, mediators may indeed improve model discrimination.  In the present study, however, the primary objective was explanatory, aimed at examining the associations between childhood obesity and neurodevelopmental delay risk within a clearly defined causal framework. For this reason, a directed acyclic graph (DAG) was used to guide variable selection and to avoid overadjustment by excluding variables positioned on the causal pathway between obesity and neurodevelopment, such as anemia.  The nomogram was developed as a clinically interpretable decision-support tool, informed by this explanatory framework rather than as a purely data-driven prediction model optimized solely for maximal discrimination. Including anemia—conceptualized as a potential mediator—could inflate apparent predictive performance by incorporating downstream information, at the cost of reduced causal interpretability and potential double-counting of effects.  We acknowledge that alternative modeling strategies focused exclusively on prediction could yield higher discrimination by retaining mediators. However, this would represent a fundamentally different objective. This distinction has now been explicitly clarified in the revised manuscript. Future studies may explore purely predictive models, including mediators, and compare their performance with the present approach. | Revised in the Discussion section (lines 626–641) and in the Limitations subsection (lines 736–742). |
| Regarding the PA indicator specifically, although the authors attempt to enhance the model by incorporating it as a key variable, there are substantial weaknesses concerning its measurement, distributional characteristics, and causal interpretation. The primary issue lies in the dichotomization of the variable. The study treats physical activity as binary—"Complies" vs. "Does not comply" with WHO guidelines —which results in a significant loss of information. By treating a child who barely meets the minimum threshold identically to a highly active child, the simple "Yes/No" classification fails to capture the gradient of intensity and movement type that is crucial in early childhood research and likely correlates with neurodevelopmental nuances.  This lack of granularity appears to have resulted in statistically anomalous data. The reported PA compliance rate is suspiciously high (95%) , which presents an epidemiological paradox when contrasted with the study's high obesity prevalence of 27%. Finding that nearly all children meet activity guidelines while nearly one-third are obese is contradictory, as low physical activity is typically a primary driver of obesity. This discrepancy strongly suggests that the measurement tool is either highly inaccurate or subject to significant social desirability bias.  Moreover, the validity of this assessment is further compromised by the age distribution of the sample. The evaluation of physical activity in children under one year old, who constitute 42% of the sample, is questionable. For an infant (<1 year), WHO guidelines focus on passive metrics like "tummy time" and "not being restrained," whereas for a 4-year-old, the guidelines involve energetic play and running. These are fundamentally different constructs. Asking a mother if a 6-month-old "meets physical activity guidelines" is cognitively more complex and prone to misinterpretation than reporting on a 4-year-old's running habits. The manuscript does not clarify how these disparate developmental milestones were standardized into a single variable. | We thank the reviewer for this thorough and insightful critique of the physical activity (PA) indicator. We fully agree that the issues raised regarding measurement, distributional characteristics, and causal interpretation are important and merit careful consideration. We address each point below and clarify how these limitations are reflected in the revised analyses and interpretation.  1. Dichotomization of the physical activity variable  The reviewer is correct in noting that dichotomization of physical activity entails a loss of information. In this study, physical activity was operationalized as a binary indicator reflecting compliance versus non-compliance with age-specific World Health Organization (WHO) recommendations. This approach was originally chosen to align with public health–relevant thresholds and to facilitate clinical interpretability in early childhood settings, where precise quantification of activity intensity and duration is challenging and caregiver-reported measures are commonly used.  However, we acknowledge that this dichotomization likely reduced variability and statistical power and may have limited the ability to detect graded associations between physical activity and neurodevelopmental outcomes. In the revised multivariable analysis, physical activity compliance was not independently associated with neurodevelopmental delay risk, and this null finding is now interpreted in light of the limited sensitivity of the binary exposure definition.  2. High prevalence of physical activity compliance and apparent epidemiological paradox  We acknowledge the reviewer’s concern regarding the very high observed prevalence of physical activity compliance (approximately 95%), particularly when considered alongside the relatively high prevalence of childhood obesity in the study population. While physical activity is only one of several determinants of obesity in early childhood—alongside dietary patterns, feeding practices, sleep, and metabolic factors—we agree that the combination of high compliance and low variability suggests a ceiling effect.  This limited contrast between exposure categories likely reduced the discriminatory capacity of the PA indicator and may have further attenuated associations in the adjusted analyses. The revised manuscript explicitly discusses this issue as a methodological limitation that may explain the absence of an independent association in the final model.  3. Measurement bias and social desirability  We agree that caregiver-reported physical activity is susceptible to recall bias and social desirability bias, particularly in healthcare settings where respondents may be aware of recommended behaviors. Overreporting of socially desirable practices is a well-recognized limitation of caregiver-reported measures in early childhood research.  While such bias may lead to overestimation of compliance prevalence, it is unlikely to be differential with respect to neurodevelopmental screening outcomes. Nevertheless, we recognize that this bias may have further contributed to exposure misclassification and attenuation of associations. This limitation has now been explicitly acknowledged and expanded upon in the revised Limitations and Discussion sections.  4. Age heterogeneity and operationalization across developmental stages  We appreciate the reviewer’s concern regarding the heterogeneity of physical activity constructs across age groups. In this study, WHO recommendations were operationalized using age-specific criteria reflecting distinct developmental expectations for infants (<1 year), toddlers (1–2 years), and preschool-aged children (3–4 years). For infants, compliance was defined based on active play and supervised prone positioning (“tummy time”), whereas for older children compliance required cumulative daily activity and, for those aged 3–4 years, inclusion of moderate-to-vigorous activity.  However, we acknowledge that harmonizing fundamentally different movement constructs across developmental stages into a single binary indicator may have introduced misclassification, particularly among infants, who constituted a substantial proportion of the sample. This heterogeneity may have further limited the capacity of the PA variable to capture meaningful differences relevant to neurodevelopment and likely contributed to the null association observed in the adjusted analyses. | Revised in the Discussion (Limitations subsection) (lines 701–730) and in the Methods section (Physical Activity Compliance) (lines 248–268). |
| Finally, there is a significant risk of reverse causality. The study identifies physical activity as a "protective factor", yet the relationship is likely bidirectional. The outcome variable (EDI test) explicitly measures gross motor skills. Children with developmental delays—the study's outcome of interest—may physically be unable to meet activity guidelines due to motor limitations such as hypotonia or coordination issues. Indeed, Table 2 reveals that in the "At Risk" group, non-compliance jumps to 30% compared to only 0.8% in the normal group. It is highly probable that their "inactivity" is a result of their developmental delay, rather than solely a cause. | We thank the reviewer for this important observation. We fully agree that, given the cross-sectional design of the study, the relationship between physical activity and neurodevelopmental delay risk is likely bidirectional and subject to reverse causality.  As correctly noted, the EDI includes domains related to gross motor development, and children classified as “at risk” may present motor limitations (e.g., hypotonia, poor coordination, or delayed motor milestones) that reduce their ability to engage in age-appropriate physical activity. In this context, lower physical activity may plausibly represent a consequence of neurodevelopmental vulnerability rather than a causal determinant.  Consistent with this interpretation, physical activity compliance was not independently associated with neurodevelopmental delay risk in the final multivariable model.  This issue has been explicitly addressed in the revised Discussion, where we state that physical activity should be interpreted as a correlational marker within a potentially bidirectional relationship, rather than as a unidirectional protective factor. We further emphasize that the temporal direction of the association cannot be established in the present study and that prospective longitudinal designs are required to disentangle causality.  To avoid causal misinterpretation, we have revised the language throughout the Discussion and Conclusions to consistently describe physical activity as being associated with, rather than causally protective against, neurodevelopmental delay risk. Accordingly, differences in physical activity compliance observed across developmental categories are interpreted cautiously and understood as potentially reflecting underlying neurodevelopmental limitations rather than causal effects. | Revised in the Discussion section (lines 604–620) and in the Conclusions section (lines 744–777). |
| In conclusion, while the initiative to create a practical tool for the early detection of developmental delays is commendable, the current operationalization of key variables significantly undermines the model's robustness. Specifically, the treatment of physical activity and the resulting high compliance rate suggest a disconnect between the data and the clinical reality of the population. Unless these methodological biases are rigorously corrected or re-analyzed, the conclusions regarding the protective factors for neurodevelopment remain open to question. | We thank the reviewer for this concluding assessment and for emphasizing the importance of rigorous variable operationalization in explanatory and predictive modeling. We fully agree that measurement choices—particularly regarding physical activity—can influence model robustness and interpretation.  In response to earlier comments, we have substantially revised the manuscript to explicitly acknowledge these limitations. We clarify that physical activity was operationalized as a caregiver-reported, age-specific indicator of guideline compliance and that the high observed compliance rate should be interpreted with caution due to potential social desirability bias and loss of granularity.  Importantly, in response to these concerns, we re-analyzed the multivariable model and revised the interpretation accordingly. Physical activity compliance was not independently associated with neurodevelopmental delay risk in the final model, and all conclusions referring to a protective effect have been removed or reformulated.  As a result, physical activity is no longer presented as a “protective factor” but rather as a correlational marker within a potentially bidirectional relationship with neurodevelopmental delay risk, consistent with the study’s cross-sectional design. The nomogram is therefore presented as an explanation-informed, clinically oriented decision-support tool intended to facilitate early risk stratification using routinely available variables, rather than as a definitive predictive model. Its moderate performance and context-specific applicability are explicitly acknowledged, and we emphasize the need for external validation and longitudinal studies to strengthen causal and predictive inferences.  We believe that these revisions adequately address the reviewer’s concerns and appropriately align the study’s conclusions with its methodological scope and limitations. | Revised throughout the manuscript. |

Reviewer #3:

Congratulations on the study. It addresses a very important topic and is developed in a very consistent way. Please find my comments below:

| COMMENT | RESPONSE | MANUSCRIPT CHANGES |
| --- | --- | --- |
| Methods:  Was a sample size calculation performed? If so, please explain it. If not, clarify how the sample was defined. It appears that all children attending the growth and development units were included; please specify this clearly.  In addition, although you clearly explained the exclusion criteria in this section, what were the inclusion criteria? Please make these explicit. | We thank the reviewer for this helpful comment regarding the definition of the study sample and eligibility criteria.  A sample size calculation was performed during the planning phase of the study to estimate the minimum number of participants required, based on the expected prevalence of the outcome of interest. During study implementation, participants were recruited consecutively, meaning that all eligible children under five years of age who attended the growth and development units or pediatric outpatient clinics at the participating EsSalud facilities during the study period were invited to participate. No random selection was applied. The final analytical sample comprised 442 children, meeting the minimum sample size estimated.  We have now made the inclusion criteria explicit in the Methods section. Specifically, the study included children under five years of age attending the participating growth and development units or pediatric outpatient clinics during the study period, whose parent or legal guardian provided written informed consent.  The exclusion criteria, which were already described, included children with previously diagnosed neurodevelopmental disorders or conditions that could independently affect neurodevelopmental assessment, such as genetic syndromes, chronic neurological or debilitating conditions, severe malnutrition, epilepsy, attention-deficit/hyperactivity disorder (ADHD), and Autism Spectrum Disorder (ASD). These clarifications have been incorporated into the revised manuscript to improve transparency and reproducibility. | Revised in the Methods section (Study Design and Population) (lines 188–215) |
| Regarding physical activity adherence, how were the data collected? Was a questionnaire used? Which specific questions were included? Please specify.  Regarding anemia assessment, did you perform laboratory analyses to obtain this information, or was it reported by caregivers? Furthermore, please describe the cutoff values used to define anemia and cite the corresponding reference (as you did for obesity assessment). Please specify. | We thank the reviewer for this comment requesting clarification regarding the assessment of physical activity adherence and anemia.  Physical activity adherence was assessed using a structured caregiver-reported questionnaire developed specifically for this study and based on the World Health Organization (WHO) guidelines for physical activity in children under five years of age. The questionnaire was administered to the child’s primary caregiver during the clinical visit. It shows age-specific questions aligned with WHO recommendations. For infants under one year of age, caregivers were asked about engagement in active play and supervised prone positioning (“tummy time”) during waking hours. For children aged 1–2 years, questions focused on total daily time spent in physical activity of any intensity, and for children aged 3–4 years, caregivers were asked about total daily physical activity including time spent in moderate-to-vigorous activities. Based on these responses, physical activity was classified dichotomously as meeting or not meeting age-specific WHO recommendations, as described in the Methods section.  Anemia status was determined exclusively through laboratory assessment. Hemoglobin concentration was obtained from venous blood samples collected as part of routine clinical care and analyzed at the institutional clinical laboratory. Anemia was defined using age-specific hemoglobin cut-off values established by the Peruvian Ministry of Health (MINSA) Clinical Practice Guideline for the prevention, diagnosis, and treatment of anemia. Specifically, anemia was defined as hemoglobin <13.5 g/dL for infants younger than 2 months, <9.5 g/dL for infants aged 2–5 months, and <11.0 g/dL for children aged 6–59 months.  Because the study was conducted in Tumbes, a coastal region located near sea level, no altitude adjustment of hemoglobin values was applied. All definitions and cut-off values were based on national MINSA recommendations, which are now explicitly cited in the Methods section.  These details have been clarified in the revised manuscript to improve transparency and reproducibility. | Revised in the Methods section (lines 248–285). |
| I suggest creating separate sections for physical activity adherence and anemia assessment in order to allow clearer and more detailed description of these aspects | We thank the reviewer for this helpful suggestion. In response, we have reorganized the Methods section and created separate, clearly labeled subsections for physical activity adherence and anemia assessment. This restructuring allows a more detailed and transparent description of the data collection methods, operational definitions, and cut-off values for each variable. These changes have been incorporated into the revised manuscript. | Revised in the Methods section (lines 179–427). |
| Did you have missing data? Please specify. If so, how were these data handled? | We thank the reviewer for this comment. Missing data were assessed prior to statistical analysis. Records with incomplete information on key variables were excluded from the multivariable analysis, and a complete-case analysis was performed.  No imputation methods were applied, as the proportion of missing data was low and no systematic patterns of missingness were identified. This approach has been clarified in the Methods section of the revised manuscript. | Revised in the Methods section, Statistical Analysis subsection (lines 349–356). |
| In the section “Maternal Knowledge of Complementary Feeding” you state: “Additional demographic variables were collected, including maternal age and educational attainment.” Please describe all demographic variables collected in the study and how they were obtained.  Also include the variables reported in the first section of the Results and in Tables 1 and 2, clarifying that they were assessed in the Methods section and explaining how they were measured. | Response:  We thank the reviewer for this comment. The Methods section has been clarified to explicitly describe all sociodemographic variables collected in the study and how they were obtained. These included the child’s age (in months) and sex, as well as maternal age (in years) and maternal educational attainment, all collected through a structured caregiver interview at enrollment. | evised in the Methods section (lines 300–306). |
| Results:  In the descriptions of Tables 1, 2, and 3, please present the cutoff values used to define obesity, anemia, and physical activity. The tables should be interpretable without the need to refer back to the Methods section. | Response:  We thank the reviewer for this comment. In response, the cut-off values used to define childhood obesity, anemia, and physical activity adherence are explicitly presented in the footnotes of Tables 1, 2, and 3.  These notes specify the diagnostic criteria applied, including BMI-for-age z-score thresholds for obesity (WHO standards), age-specific hemoglobin cut-off values for anemia (MINSA guidelines), and age-specific WHO definitions for physical activity compliance. As a result, the tables are fully interpretable as standalone elements without the need to refer back to the Methods section. | **Revised in the Tables section (lines 1187–1258).** |
| Discussion:  In the first paragraph, it is not necessary to restate the study objective. The rest of the paragraph describes results rather than providing discussion. Please review this.  In which contexts do you consider the use of the nomogram applicable? Please expand the discussion regarding the external validity of its use. | We thank the reviewer for this valuable comment. The Discussion section has been revised to avoid restating the study objective in the opening paragraph and to strengthen its interpretative focus rather than reiterating descriptive results.  In addition, we have expanded the Discussion to explicitly address the contexts in which the proposed nomogram may be applicable. We clarify that the nomogram is intended as a decision-support tool for risk stratification in primary care and community health settings, particularly in low- and middle-income contexts where access to specialized neurodevelopmental assessment is limited. We also emphasize important considerations regarding external validity, noting that differences in socioeconomic conditions, healthcare access, and early stimulation environments may affect model performance. Accordingly, we highlight the need for external validation and potential recalibration before applying the nomogram in populations outside the EsSalud healthcare network. | Revised in the Discussion section (lines 518–742). |
